# Social Policy and Cognitive Enhancement: Lessons from Chess

**DOI:** 10.1007/s12152-018-9354-y

**Published:** 2018-02-22

**Authors:** Emilian Mihailov, Julian Savulescu

**Affiliations:** 1 Research Centre in Applied Ethics, Faculty of Philosophy, University of Bucharest, Splaiul Independentei no. 204, 060024 Bucharest, Romania; 2 Oxford Uehiro Centre for Practical Ethics, University of Oxford, Suite 8, Littlegate House, St Ebbes Street, Oxford, OX1 1PT UK

**Keywords:** Cognitive enhancement, Mind sports, Chess, Cognitive complexity, Cognitive expertise, Policy, Trustworthiness

## Abstract

Should the development of pharmacological cognitive enhancers raise worries about doping in cognitively demanding activities? In this paper, we argue against using current evidence relating to enhancement to justify a ban on cognitive enhancers using the example of chess. It is a mistake to assume that enhanced cognitive functioning on psychometric testing is transferable to chess performance because cognitive expertise is highly complex and in large part not merely a function of the sum specific sub-processes. A deeper reason to doubt that pharmacological cognitive enhancers would be as significant in mind sports is the misleading parallel with physical enhancement. We will make the case that cognitive performance is *less mechanical* in nature than physical performance. We draw lessons from this case example of chess for the regulation of cognitive enhancement more generally in education and the professions. Premature regulation runs the risk of creating a detrimental culture of suspicion that ascribes unwarranted blame.

## Introduction

At the 2008 Chess Olympiad in Dresden, officials requested that Vassily Ivanchuk submit to a drug test after losing to Gata Kamsky. The media reported that “he stormed out, kicked a pillar in the lobby, pounded a countertop in the cafeteria with his fists and then vanished into the coatroom.”^1^ Perhaps this was adding insult to injury, angering an exhausted and volatile grandmaster. But since Ivanchuk refused to take a urine test, the officials treated his refusal as a confirmation of doping.

The decision caused outrage among chess professionals. Many elite players believe that doping accusations are an insult to their profession and argue that performance enhancing substances bring no benefits to the sport. In an open letter of support of Ivanchuk, grandmaster Alexei Shirov accused the World Chess Federation (Federation Internationale des Echecs, known as FIDE from its French acronym) of unjustifiably imposing anti-doping rules just to meet the requirements of the International Olympic Committee for labelling chess as an Olympic sport.^2^ In response, the FIDE President Kirsan Ilyumzhinov argued that anti-doping rules were necessary to protect chess against cheating:

“Chess as a sport itself deserves the clean competition of the players, devoid of falsifications, cheating and doping. From the very beginning, we were well aware of that in chess we would not have to cope with steroids or other hormones used in the physical sports, but at the same time the scientific research identified several substances that could affect the mental performance of a chess player.”^3^

The outrage of elite chess players may be motivated by professional integrity,^4^ but there is a tension between the scepticism of professional players and the belief of FIDE that pharmacological cognitive enhancement could offer advantages. Scepticism about enhancement may be driven by assumptions equating doping to physical, mood, and motivational enhancement.^5^ But this misses the germane challenge of pharmacological developments which are shown to have beneficial effects directly on basic cognitive functions.

Due to the expansion of prospective technological and pharmaceutical enhancements, the use of performance enhancing substances in sports is fiercely debated. However, much of the debate has focused on physical sports, while discussions about cognitive enhancement have focussed on risk, benefit and the public interest, particularly in relation to education and employment [2, 3]. Now, there is a growing tendency to take the development of enhancement substances as providing means of mental doping in cognitive sports. The International Mind Sports Association, which regulates bridge, draughts, go, xiangqi, also adheres to the World Anti-Doping Agency’s policy on prohibited substances. Should the development of smart drugs for the general population outside therapeutic contexts raise worries about doping in cognitively demanding sports and games? More generally, should cognitive enhancement be banned in complex professions or education?

There are reasons to resist this tendency, at least for the present time. In this paper, we use the test case of chess and we argue that a ban is based on a misunderstanding of the evidence about enhancement, the general connections between cognitive processes and the complexity of mental performance. Cognitive enhancement substances should, nevertheless, be monitored, just as caffeine and codeine are and further evidence gathered. Ethical analysis will be necessary to evaluate this evidence and decide whether cognitive enhancement is morally permissible, but in the first instance we argue that it is a mistake to jump to conclusions about pharmacological enhancement of complex cognitive performance, as in the case of chess, from evidence of modest improvements outside that activity or in lay people. We will argue that there is a stark discrepancy between the high expectations and the actual enhancement effects. The realistic picture we outline on the effectiveness and *modus operandi* of pharmacological cognitive enhancement will help assess whether there is direct and indirect evidence for an impact on performance in chess. We further argue that we should avoid assuming that enhanced cognitive functioning on psychometric testing is transferable to chess performance because cognitive expertise is highly complex and in large part not merely a function of the sum specific sub-processes. A deeper reason to doubt that pharmacological cognitive enhancers would be as significant in mind sports is the misleading parallel with physical enhancement. We will make the case that cognitive performance is *less mechanical* in nature than physical performance.

If we are right, the case of chess provides important lessons to be learned for the broader social context of using cognitive enhancement. We cannot form policy on complex questions about cognitive performance in competitive activities, education, or the workplace from simple lab tests that have questionable ecological validity. Premature regulation runs the risk of creating a detrimental culture of suspicion that ascribes unwarranted blame.

## Belief in Enhancement

According to the Chess WADA Anti-Doping Policy, the most relevant banned substances for chess are amphetamine derivatives (Adderall, Ritalin), ephedrine and methylephedrine, pseudoephedrine, and Modafinil. Notably, caffeine and codeine are not prohibited, but figure in the Monitoring Program.^6^ The reasoning is probably that a normal dosage of caffeine or codeine is highly unlikely to present significant benefits to players. The FIDE believes that cognitive enhancers have the potential to be of benefit in chess, and has thus implicated Modafinil, Adderall and Ritalin. In therapeutic contexts, Modafinil is used to treat narcolepsy, shift work sleep disorder and excessive daytime sleepiness, by improving wakefulness. Ritalin and Adderall are nervous system stimulants used for ADHD treatment, a disorder characterised by concentration and difficulty with impulse control.

Many studies have shown that Modafinil, Adderall and Ritalin benefit people in non-therapeutic contexts. Modafinil enhances performance on tests of digit span, visual pattern recognition memory, spatial planning and stop signal reaction time [4, 5]. Additionally, there is evidence that non-sleep-deprived individuals given Modafinil have higher accuracy and marginally faster response times on tests of conceptual ability [6]. Administration of methylphenidate (Ritalin) has been shown to improve problem solving abilities, spatial working memory, declarative memory consolidation and the ability to divide and relocate attention [7–9]. Equally important, stimulants and Modafinil have significant non-cognitive effects on motivation, mood and alertness, factors which are closely related to overall cognitive performance [10].While many professional players may not be aware of these kinds of effects, FIDE has used such evidence as a basis to ban cognitive enhancers.^7^

The evidence used by FIDE refers to enhancement effects outside mind sports, but a recent study documents for the first time enhanced performance in chess by methylphenidate and Modafinil. In randomized crossover trial, Andreas Franke and his colleagues [11] administered Modafinil, methylphenidate and caffeine or placebo to participants who played against chess software. Results showed an increased performance in average scores for all three substances, implying that the players performed 3–4 percentage points above placebo performance. We will discuss below how relevant this evidence is and whether it is sufficient to justify a ban.

Another potential cognitive enhancer in chess is transcranial direct current stimulation (tDCS). TDCS stimulates the brain by passing a low intensity current through two electrodes placed over the head which modulates neuronal activity. A recent meta-analysis revealed a small but significant effect in healthy participants of left dorsolateral prefrontal cortex stimulation coupled with working memory training [12]. Such devices are widely used in computer games [10]. They are not prohibited.

## Effectiveness of Pharmacological Cognitive Enhancement

The media uses metaphors like “miracle drug” and “brain steroid” to describe cognitive enhancers [13]. These contribute to an exaggerated image of the effectiveness of enhancers. Part of the debate has misleadingly focussed on extreme forms of bioenhancement that promise radical transformations of human capacities, especially drugs that might dramatically increase intelligence to super-human levels, the prospect of bionic athletes, and biotechnological interventions that would extend our life span indefinitely [14–16]. In response, some have argued that there is a need to refocus on normal range human enhancement [17]. As we will argue, there is a stark discrepancy between the high expectations and the actual effects.

Overall, no sweeping general conclusion can be drawn regarding the effectiveness of pharmacological cognitive enhancement even in the laboratory setting. There is no general effect exhibited by smart drugs, as each drug in its own right and dosage will have different effects [18]. In their meta-analysis of randomized controlled trials of Methylphenidate, Repantis et al. [19] found some effect on memory, but no significant improvements in attention or executive function. In other experiments, Methylphenidate did not affect spatial working memory or planning [8]. Farah et al. [20] confirm that Methylphenidate and amphetamine improve memory when the retention interval between study and test was longer than an hour, but the evidence on executive functions was inconclusive. Fond et al. [21] identified enhancement effects of Methylphenidate on working memory, but again no significant effect on attention and executive functions.

With respect to Modafinil, the reviews of Fond et al. [21] and Repantis [22] found moderate positive effect on attention, but none for memory. Other reviews conclude that although there is clear evidence of enhanced executive function and memory in sleep-deprived individuals, for well rested individuals there is a large number of null results and occasionally even cognitive impairment [20]. Battleday and Brem [23] also highlight that most studies did not report any effect of Modafinil intake on problem-solving abilities, and a few even reported impairments in divergent creative thinking. It seems that Modafinil preferentially affects attention and executive function of lay population, with no significant influences on memory and problem-solving abilities.

It is also important to highlight that enhancement effects are not always upgrades over normal performance. Studies which measure cognitive performance do not employ the same enhancement model, raising methodological problems in extrapolating from overviews. We should distinguish between the ‘better than baseline’ model, used to measure improvements in normal functioning, and ‘sustaining the baseline’ model, used to measure the extent to which normal functioning can be preserved in adverse conditions. Modafinil was mainly tested in studies with sleep-deprived individuals [22], that is, sustaining the baseline.

The high expectations of the potential of pharmacological cognitive enhancement fuelled by the media and popular culture^8^ should be tempered. Much of the cognitive enhancement scientific literature is 15 to 20 years old and that more recent investigations are often equivocal. Current evidence suggests only modest effects on some cognitive sub-functions measured by fairly simple specific laboratory tasks, with no effects on other cognitive sub-functions and even impairments. Farah et al. [20] refer to this pattern of limited improvements on some specific tasks and impairment on others as being “familiar” for pharmacological cognitive enhancers.

Enhancement effects are modest in healthy individuals, but even this size can be misleading because it discards the variations within subgroups. If we look into subgroups whose baseline performance is poorest the effects become more significant. In many studies, Methylphenidate improved working memory in people who were low performers, though on the other hand, it impaired performance in participants with high working memory baseline [24, 25]. There is also evidence that Modafinil is less effective in high-performing individuals than in low-performing individuals [26], as well as in high-IQ versus low-IQ individuals [4]. These results suggest another “familiar” pattern for pharmacological cognitive enhancement – more consistent improvements in low performing individuals and no significant influence or even impairment on individuals with a high performance baseline. It is, importantly, possible that putative cognitive enhancers *impair* certain functions in elite chess players.

To get an estimate about the size of some pharmacological enhancement effects, naps are more effective in maintaining performance than Modafinil and amphetamine during periods of sleep deprivation in healthy individuals [27]. The more realistic picture we outlined about the effectiveness and *modus operandi* of pharmacological cognitive enhancement helps to draw more reliable implications for professional chess performance.

## Implications for Chess Performance

### Direct Evidence?

We have argued that the existing evidence of enhancement effects on cognitive functions is modest in general, and there is the possibility of impairment in certain domains. Moreover, there is no direct evidence relating to elite professional chess performance. Almost all relevant evidence is taken from studies on healthy lay subjects performing basic psychometric tests.

There is one recent study showing enhancement effects on chess related tasks, but subjects were in fact amateurs, which questions its ecological validity for professional performance. What is striking in this study is how the enhancement effect was supposedly mediated. It was not improved memory, accuracy or planning, but what Franke and his colleagues described as “more reflective decision making” ([11], p. 257). The average reflection time per game was considerably higher compared to placebo during middle game (moves 11 to 25). It seems plausible that “more reflective decision making” did not make players “deeper” thinkers, as media outlets claimed.^9^ Rather, it seems that the effect was to delay making a move. Subjects just took more time for game analysis, presumably having the subsequent effect of seeing more optimal moves and counter-moves, while players in the placebo condition moved faster, without thoroughly assessing the positions. Improvements were not *direct outputs* of enhanced thought process, but rather *side-effects* of a better impulse control of the tendency to react quickly to the opponent’s moves.^10^ It is standard training to teach beginners to take their time before each move, as they move quickly without thinking too much, but this is unlikely to help elite professionals who have in depth experience in allocating time to evaluate complex positions. Such professionals can literally spend hours deliberating over position and possible moves.

For these reasons, conclusions about pharmacological cognitive enhancement as a form of performance enhancement in cognitive sports should not follow directly from an analogy with physical performance enhancement. In the case of essentially physical sports, the evidence points to strong enhancement effects in directly relevant activity and is taken from studies with elite and sub-elite athletes performing their specific sport (see for example [28]). Moreover, it is a mistake to draw conclusions about complex cognitive activities like chess directly from mild cognitive enhancement effects in a diverse population of lay people on simple psychometric tasks.

To advance the debate on mental performance, major research needs to start with the influence of cognitive enhancers on elite and sub-elite chess players performing specific chess tasks. We should aim for a minimal ecological validity. In chess there is a clear system of rating how players excel, heavy opening theory, well organized chess puzzles based on difficulty levels, and clear standards of strategic thinking. Tests for measuring performance at chess specific tasks could be devised with regards to long term memory, pattern recognition and strategic thinking, taking into account, as well, the strength of players.

Even under strict laboratory conditions, enhancement effects are generally minor and often the same as with conventional means. The single existing chess study showed caffeine to be just as effective as Modafinil and was slightly better than methylphenidate [11]. So even if we get the same results for professional chess players as it is documented for lay people on tests of opening moves, we should expect minor effects as well, which could more easily fade away outside experimental conditions. Moreover cognitive enhancers like methylphenidate and Modafinil appear to offer little advantage over caffeine, which is already legal.

### Indirect Evidence?

One objection to our argument that we cannot infer significant benefits to chess from current evidence is that pointing to lack of direct evidence does not rule out the possibility that cognitive enhancement is probably achievable in an indirect manner [29]. Pharmacological substances improve cognitive sub-functions such as attention, alertness, memory and information processing. These cognitive sub-functions are used to support general cognitive abilities. These more integrated cognitive processes determine the way in which complex tasks of chess playing are performed. For example, pharmacological enhancement improves memory, a cognitive function which can improve the learning process. Then, improvements in learning will positively support the task of solving more chess puzzles. The argument assumes that improvements at the level of cognitive sub-functions will translate to a higher level of cognitive functioning and, most decisively, the improvements of the latter will translate to domain specific and highly demanding cognitive processes.

While this objection may have some plausibility, it is possible to argue equally in the opposite direction. As complexity grows, it is plausible to assume that improvements on basic memory tasks will taper off, after which even impairments can be seen. After all, since memory improvements are minor, why shouldn’t we assume that enhancement effects will phase out as we work using more integrated and complex cognitive processes? Maybe improvements are seen precisely because task performance does not require exceptional and integrated cognitive processing. By using an exceptionally complex visual attention task, Rogers et al. [30] concluded that methylphenidate actually *disrupts* the allocation of attention between relevant and irrelevant features of the environment. So we might indirectly infer that cognitive enhancers might impair performance in chess. Another study using a complex video game, which requires problem-solving strategies, found that Ritalin (methylphenidate) disrupted performance [31]. In short, results of putative enhancements on integrated cognitive processes are generally negative [32].

Another version of this objection is that, while there is no general effect of pharmacological enhancement on complex processes, we should distinguish different substances. Stimulants (such as Ritalin and Adderall) seem to preferentially target memory with no significant effects on other cognitive functions, while Modafinil seems to preferentially target attention and executive function with little or no significant effect on memory. This implies that no sweeping claim can be made about indirect enhancement. At best it can be argued that a *particular* substance can preferentially target a *particular* cognitive function. Some tasks may rely more on memory, while others may be based more on executive function. We should not assume general correlations between all pharmacological cognitive enhancers and all mind sports.

Moreover, the relation between a *particular* substance and a *particular* cognitive function should be determined more precisely. It is not enough to argue in favour of enhancement effects from the premises that a particular substance improves memory and that better memory improves chess playing. Memory and executive function have many sub-domains, which in psychometric tests are measured independently from each other. A pharmacological enhancer does not improve memory in general, but specific sub-functions and not all memory sub-functions improve performance at chess. Suppose that a particular substance has significant enhancement effects on visual memory. There is in fact evidence that visual memory is relatively unimportant to chess skill ([33]), whereas storage in long term memory is central [34].^11^

However, a “familiar” pattern of pharmacological enhancement that we have highlighted is characterised by more consistent improvements in low performing individuals and no significant influence on individuals with high performance baseline or even impairment. Chess is a highly demanding mental activity and, not surprisingly, its practice can be a useful tool to enhance our intellectual abilities. In light of this, there are correlations between chess skill and IQ [36, 37]. Also, elite players possess very strong long-term memory abilities. Chase and Simon [34] showed that the differences in ability of chess players to recall positions are explained by the experts’ storage of thousands of chunks of information in their long-term memories. Others estimate that one needs to memorize about 100,000 opening moves in order to reach high levels of expertise in chess [38]. It is unlikely that we will find professional chess players in the low baseline group, where the benefits of pharmacological enhancement are to be found. Moreover, there is no evidence of any cognitive enhancer affecting long term memory at this level.

The other “familiar” pattern of pharmacological enhancement is exhibited by limited improvements on some specific tasks and impairment on others, calling into question the status of the overall enhancement even of specific enhancers. This undermines the idea that improvements of a particular function will bring benefits to more highly integrated cognitive processes. Enhancement effects are also coupled with impairments of other particular functions, which may reduce the beneficial effects or even de-enhance cognitive performance. A few studies reported impairment of problem-solving abilities after taking Modafinil. And most importantly, although Modafinil and methylphenidate increased reflection time per chess game in the only ecologically valid study of chess performance, resulting in more wins, it also led to more losses due to running out of time as compared to placebo [11].

Here is one example of a potential pharmacological enhancer which we did not mention in our analysis, but nevertheless, it is famously related to chess performance and nicely illustrates the point. The use of beta-blockers is banned in archery, billiards, snooker. Beta-blockers can inhibit unwanted influences of arousal, anxiousness, or stress, by blocking adrenaline from activating a “fight-or-flight” response. It is shown that low rated and sub-elite chess players who exhibit heart rates in excess of 200/min and large increases in catecholamines tend to make more simple mistakes [39]. The negative effects of adrenaline on the quality of play can be diminished by reducing the players’ heart rates and anxiety. On the other hand, one famous self-experiment conducted by Helmut Pfleger, a Grandmaster and medical doctor, tells us the impairment side of the story. Pfleger tested the effects of beta-blockers on himself in 1979, in a match with former world chess champion Boris Spassky, by taking a tablet of Beloc before the game. His heart rate went down to 40 to 65 (in comparison with his normal range of 80–110) and he lost almost immediately to Boris Spassky, after just twenty moves. Pfleger stated that his game collapsed when his blood pressure plunged, drawing the harsh conclusion that both mentally stimulating and mentally calming medication have too many negative effects [40].

Even though beta-blockers can enhance performance of low rated and sub-elite players when a massive release of adrenaline chokes the functioning of cognitive skills, it is not clear whether elite chess players can benefit. At least one elite player on one occasion did not. Beta blockers may reduce the probability of making simple mistakes which are facilitated by anxiety. However, when it comes to fine-tuned abilities of elite players, the prospect of fatal impairment is present. Mentally calming medication can decisively numb elite players’ ability to foresee potentially dangerous patterns and react accordingly. This simple beta blocker “experiment” is of course not a randomised controlled trial but it is does suggest the potential for enhancers to be damaging to elite performance.

### General and Specialized Cognitive Abilities

The mind is not based on a delicate interdependence of cognitive functions, such that one intervention will alter “something throughout all parts of the immeasurable whole”, to use the expression of German philosopher, Johann Gottlieb Fichte.^12^ Cerebral hemispheres work together to maintain mental unity, as shown by split-brain studies [42], but the mind also contains a conglomeration of independent systems. Announcements of enhancements effects on memory, learning or reasoning are based on imprecise general labels of cognitive functions. Undifferentiated talk about cognitive enhancement is misleading without discussing which particular cognitive function is investigated. For example, memory is not a monolithic brain system, as one has to take into account the distinctive neurobiological and neurochemical systems that make up, at the very least, semi-dissociable systems of memory [43], such as long and short term memory. This point will help to highlight that cognitive complexity also consists in developing and sustaining specialized cognitive abilities, which are relatively independent from general thought processes.

Professional chess players are usually perceived as having superior intelligence, abstract thinking and memory abilities. This might be true to some degree, but it is a misleading picture. Between experts and non-experts there aren’t significant differences in their general thought processes [44]. Master level players consider almost the same number of possible moves, search heuristics or engage in the same depth of search as weaker players. When required to recall positions with chess pieces that are randomly placed on the board, expert players have the same short-term memory as non-experts.

What then makes the difference if not the gross characteristics of thought and memory processes? There is a vast body of data which explains cognitive expertise by acquiring a large, complex, and flexible amount of knowledge during domain-specific practice and experience ([34], [45–47]). With extensive practice a specialized content determined by a set of rules is stored in long term memory. Experts are then able to identify, select and combine chunks of information that are relevant for their cognitive tasks. For example, expert chess players do not engage in more intense cognitive processing than non-expert players, but they are far better at identifying specialized patterns. In comparison, non-experts consume cognitive and time resources analysing irrelevant content [34].

In general, non-experts do a poor job at discarding irrelevant information, whereas experts master the ability to process the meaningful information for a specific domain. However, the playing field is levelled when cognitive tasks involve information and patterns outside the specific domain. As noted earlier, chess players do not perform better than non-experts at recalling non-specialized information. Counterintuitively, nor do super-memorizers. In one study, Ramon et al. [48] had the opportunity to test two subjects with exceptional success in the World Memory Championships. As expected, super-memorizers easily out-performed control subjects at face-name learning tasks similar to those used in international memory competitions. However, the playing field was levelled when all subjects performed a task outside the specialized domain. Despite their superior performance at specialized tasks, super-memorizers did not differ from control subjects in non-specialized tests, such as face inversion recognition.

It seems that general cognitive abilities are considered for the most part to be unrelated to expertise. Against the popular picture, Gobet et al. note that there is not “a single study that has shown that more skilled chess players outperform less skilled chess players on any psychometric test.” ([33], p. 305) Others point out that general intelligence is “either unrelated or weakly related to performance among experts (…); factors reflecting motivation (…) are much better predictors of improvement” ([45], p. 280).^13^ One should avoid assuming that enhanced cognitive functioning at psychometric tests is simply transferable to performance of cognitive expertise.

## The Mechanical Model of Enhancement

We pointed out earlier that judging pharmacological cognitive enhancement as a form of performance enhancement in cognitive sports should not follow the example of physical enhancement substances because the levels of relevant evidence are not even close. However, there is a deeper reason as to why cognitive performance may not parallel physical performance. Physical performance is *more mechanical* in nature than cognitive performance. Enhancement effects on physical performance are more quantifiable, robust and significant, because the causal links of improvements are more straightforward between enhancement interventions and single physiological mechanisms of direct relevance to performance.

The mechanical model holds that if we improve a specific mechanism, or component of performance, we will likely improve overall performance. This model is well illustrated in endurance sports. Endurance athletes benefit from increased haemoglobin levels, which increase their oxygen carrying capacity. Such improved oxygen delivery improves athletes’ aerobic capacity, defined as the maximum amount of oxygen that can be consumed by the body per unit time. A variety of methods are used for blood manipulation, from blood transfusions, administration of Erythropoietin (EPO), to living or training at high altitudes. It is said that “increasing the oxygen transport capacity of the exercising skeletal muscles, either by means of training or doping, is the most powerful tool for improving athletic performance in aerobic sports.” ([50], p. 837) The objective is to deliver more oxygen to muscles in order to significantly improve time to exhaustion or race times.

Several studies have documented the enhancement benefits of blood manipulation. Three weeks of living and training at 1380 m and simulated altitude exposure at 3000 m can improve time to exhaustion by 9% in comparison to baseline [51]. It has previously been observed that autologous blood transfusion improved 10,000 m running performance by approximately one minute in six highly trained male distance runners [52]. Williams et al. [53] demonstrated that autologous blood transfusion improved 5-mile treadmill run times by 44 s, with reduced self-reported perceived exertion. A significant fall has also been demonstrated in the race times of cross-country skiers. The significantly increased performance was observed both 3 h and 14 days after reinfusion [54]. Following EPO administration, trained subjects maintained a faster pace throughout a 3000 m time trial compared to baseline, with running performance improved by approximately 6% [55].

While there are other factors which can be critical to performance, such as anaerobic thresholds, exercise and resource efficiency, and athletes still have to train hard, be fit and fast in order to win, the link between oxygen carrying capacity and endurance performance is clearly established and intimately related. The mechanical model of enhancement refers to such direct causal links between well determined single physiological functions and performance. Some causal links will be more relevant to sprinting, while others to endurance. These mechanical links are still subject to fine-tuned interventions of dosage and range. For example, increasing muscle mass over some threshold can throw off balance or impair agility. Our point is not primarily about the right balance or how many factors interact in order to sustain performance, but rather about dissociable physiological functions that are of direct relevance to performance, which indeed do not necessarily transfer into performance improvements in adverse conditions or sub-optimal interaction. We claim that in principle one could identify a mechanical basis of performance, based on direct causal links between physiological functions, at least in endurance sports, all things being equal. The mechanical model does not hold for all physical sports, especially those in which neurological systems contribute in an essential manner to performance. For physical sports in which the practice of a skill is central, such as balance and calculating appropriate body position in successful landing in gymnastics, it would be very difficult to determine a causal link between enhancement interventions and single physiological mechanisms. Notwithstanding, the mechanical model of enhancement is applicable to many sports where dissociable physiological functions contribute to a great extent to performance.

Cognitive performance in mind sports (chess, go, bridge, draughts, xiangqi), on the other hand, cannot be equated to relatively straightforward links between enhancement interventions and single neural mechanisms. Although it has been established that dopamine, which is modulated by methylphenidate, plays a role in reinforcement learning in response to rewards, Husain and Mehta [25] conclude that “simple conceptualizations linking a specific neurotransmitter to a single cognitive function are unlikely to be helpful.” Mental performance, especially in cognitive sports, is more complex than mapping causal links between precise locations in the brain and particular cognitive functions. Targeting single aspects of cognitive mechanisms has even proven in some cases to have downsides. We have described how Ritalin disrupts performance in a complex video game, Modafinil can contribute to players losing chess games due to running out of time and mentally calming medication can cause chess performance to collapse.

Thus, we should not think of enhancement in demanding and specialized cognitive activities in terms of a mechanical model as illustrated by physical endurance performance. It might be the case that interventions will have to work more *holistically* on cognitive functioning to prove useful, or on more complex, yet understood, specific domains. Future exploration could shed light on this by comparing, for example, the effects of pharmacological substances and brain stimulation by magnetic fields (TMS) or electrical currents (tDCS), as the latter could influence brain areas and the neuroplasticity of the brain as a whole.

## Normative Implications: The Cassandra Problem

It is unlikely that modest improvements on basic psychometric tests constitute mental doping, understood as enhanced performance in demanding cognitive activities. The possibility of mental doing deserves a sophisticated analysis which draws on interdisciplinary insights about how the mind works, especially in the context of cognitive expertise.

Another important and troubling part of the story about how the enhancement evidence has been misused is its insidious moral cost. The world of sports has been shaken by far too many examples of misconduct. There is evidence of widespread doping across many sports including athletics, tennis, football, and cycling. In conditions of deepening distrust, it is rational to be sceptical and devise ambitious policies to increase compliance. However, focusing only on examples of misconduct, we lose sight of what has been called “the Cassandra problem” [56]. Apollo tried to seduce Cassandra, the beautiful daughter of King Priam and of Queen Hecuba of Troy, by endowing her with the power of prophecy. When Cassandra refused him, Apollo cursed her that nobody would ever believe her prophecies. Even though she warned the Trojans about the Greeks inside the Trojan Horse, she could not do anything because no one trusted her. For us, the Cassandra problem is that of misplaced mistrust and unwarranted suspicion.

We are unreasonable in adopting the suspicious perspective when we go against the evidence. While ambitions of having doping-free sports may seem attractive, a default of a sceptical position is not in itself superior. As Onora O’Neill puts it, “Blanket scepticism may sound more sophisticated than blanket credulity, but has no more to commend it” (2002, p. 141). If we want to have well placed suspicion in mind sports, then we should not settle for the current evidence of cognitive enhancement in general. Pharmacological enhancement is characterised by more consistent improvements in low performing individuals and no significant influence on individuals with high performance baseline or even impairment. Further, it is characterised by improvements on some specific tasks and impairment on others, calling into question the status of the overall enhancement. Cognitive complexity consists in developing and sustaining specialized cognitive abilities, which are relatively independent from general thought processes, usually targeted by pharmacological cognitive enhancement. Cognitive performance does not have a mechanical basis of causal links between enhancement interventions and single neural mechanisms. Devising social policy without an adequate understanding of cognitive complexity is unwarranted and could lead to misplaced mistrust, and consequently to an artificial culture of suspicion, as appears to have happened in the Ivanchuk case.

It could be objected that trust may also be undermined by not implementing a ban.^14^ After all, cognitive enhancers *might* work and chess players don’t need them. However, our argument does not imply that a policy banning cognitive enhancers cannot be overall justified, nor that its costs could not in principle be compensated against greater benefits. Rather, our point is that prohibitive policies are not morally neutral with regards to undermining trustworthiness. Many people intuitively believe that we promote trustworthiness by prohibiting some practices or substances. While this may be so when good evidence is available, and policies can be effectively policed, placing many competitors under suspicion in spite the evidence could undermine the game. The Cassandra problem reveals a moral cost which cannot be ignored simply by hoping that creating a climate of suspicion erratically will lead to greater trustworthiness in the end.

We should consider the cost of misplaced mistrust and, consequently, look for better means of reducing it. For example, a monitoring policy could be more suitable until we see increased usage of pharmacological cognitive enhancement and more evidence gathered. Our analysis suggests several standards of evidence which are relevant for ethical analysis to decide whether cognitive enhancement is morally permissible.

Firstly, the one study involving the use of cognitive enhancers used amateur players, it showed modest effects which were reversed on subsequent games and the effects were no greater than caffeine. What is clearly needed are ecological studies of elite players over multiple games, comparing these effects to caffeine and other accepted enhancement practices. If Modafinil and Ritalin are no more effective at improving chess performance than other enhancement substances like caffeine, there are no good reasons to ban them since caffeine is freely permitted and these substances are no more dangerous than caffeine. Further relevant evidence could be gathered by comparing thresholds between traditional and pharmacological enhancers, not only between currently permitted and prohibited substances. In one recent overview, non-pharmacological enhancement practices, such as naps, were found to be as effective as pharmacological enhancers such as Modafinil, methylphenidate and caffeine [57].

Secondly, if we want to get more reliable indirect evidence of enhancement in chess, we should focus on accurate models of such activities, like storage and retrieval from long-term memory of domain specific chunks of information and how this relates to pattern recognition. Talk about cognitive enhancement effects in general should be avoided because it encourages the misperception that enhancement effects are spillover effects. There are various and distinctive specialized processes that make up the panoply of cognitive expertise, which is considered for the most part to be unrelated to general cognitive abilities. We require research into specific enhancement effects of different cognitive enhancers on specialized cognitive skills or holistic effects on specific domains. While clearly it is too high a standard to require placebo controlled randomised trials involving elite players at tournament, it is not too high a standard to improve on existing research. Placebo controlled trials involving elite players, instead of amateurs, in laboratory conditions against computers would be a reasonable level of evidence to require. If this showed greater improvements than were achieved by caffeine and napping, then a ban on grounds of “significant” performance enhancement would be warranted.

## Conclusion: Lessons from Chess for the Regulation of Cognitive Enhancement

Chess is the paradigm of a complex integrated cognitive activity which can be played in highly competitive environment. It is not clear whether cognitive enhancers confer an advantage (or disadvantage) in chess, and what the nature and extent of that advantage might be. There is no reason based on current evidence to infer a significant probability of providing performance enhancement effects in chess. Evidence of cognitive enhancement in general is too blunt and misleading. We need, instead, to conduct ecologically valid experiments on the benefits and risks of cognitive enhancement on accurate models of such activities. The current evidence documents modest enhancement effects at basic psychometric tests based on models of general thought processes. However, mental performance, especially in contexts of cognitive expertise, is more complex than mapping causal links between precise neural circuits and particular cognitive functions. Enhancement interventions would have to be more sophisticated than the mechanical model, which assumes a straightforward influence between well determined single mechanisms and performance. Mental performance is not a monolithic concept. In contexts of cognitive expertise, performance is based on distinctive specialized cognitive abilities that are relatively independent from general intelligence.

Cognitive enhancement is becoming more common in education and might also seem attractive to professionals to improve focus, wakefulness and performance, when fatigued ([58], [59]). Duke University banned the use of cognitive enhancement on campus.^15^ Such a move is, in our view, premature. We should attempt to derive evidence involving relevant complex tasks which are based on accurate models of such activities. Learning and professional work are complex activities similar to chess. We cannot expect to read off from even modest improved performance at a simple laboratory task, a gain in a complex activity such as problem solving. It is necessary to identify what are the distinctive or specialized cognitive abilities in academic learning and particular cognitive demanding professions, and determine how well the *modus operandi* of current enhancement interventions matches with what the sciences of the mind tell us about cognitive expertise in these domains.

Ethical analysis will surely be necessary to decide on a rational policy on cognitive enhancement. But in the first instance, we need ecologically valid scientific research into the nature and magnitude of effect on complex cognitive tasks. The lesson from chess is do not jump the gun. As in chess, think before you move.

## Footnotes

1
http://www.spiegel.de/international/zeitgeist/outrage-over-ivanchuk-the-great-chess-doping-scandal-a-595819.html

2 See the press release here: http://en.chessbase.com/post/alexei-shirov-let-us-ban-fide-

3
http://gambit.blogs.nytimes.com/2008/12/26/world-chess-federation-president-addresses-controversies/?_r=0

4 According to the WADA [1] Anti-Doping Rule Violations (ADRVs) Report, there were no anti-doping rule violations in chess.

5 After the Ivanchuk scandal, many top level grandmasters were asked by the media what chess doping means to them. Here are some typical answers: “I think, chess is more intellectual than physical kind of sport. There is no point in speaking about chess doping.” (Peter Leko) “I think doping doesn’t assist chess-players that much and I doubt if anybody uses it at the top-level tournaments. Chess isn’t sport in its original scope, it’s not an Olympic kind of sport like track-and-field or gymnastics.” (Shakhriyar Mamedyarov) See: https://chessdailynews.com/whats-chess-doping-to-you/

6 The WADA Prohibited List and Monitoring Program can be found at: http://list.wada-ama.org/

7 See the FIDE Chess WADA – Anti-Doping Policy, Nutrition and Health at http://www.fide.com/FIDE/handbook/WADA%20Anti%20Doping.pdf

8 The film Limitless portrays an exaggerated version of the influence of Modafinil.

9 See https://qz.com/905546/smart-drugs-like-modafinil-and-ritalin-can-be-used-as-doping-agents-to-enhance-performance-in-chess-games/

10 The study has an important limitation in this regard. It did not assessed participants for ADHD symptoms such as inattention, hyperactivity or impulsivity during the study.

11 Similar findings show that other cognitive sports, such as bridge, are supported by working memory, but not by semantic memory [35].

12 In his *The Vocation of Man*, Fichte says that one “could not remove a single grain of sand from its place, without thereby (…) altering something throughout all parts of the immeasurable whole”. ([41], p. 26)

13 This suggests that motivation enhancement could potentially be more effective than cognitive enhancement on chess performance. As for ethical implications, some studies document that lay people consider motivation enhancement as less morally wrong than cognitive enhancement [49].

14 We thank one anonymous referee for this objection.

15 The policy can be consulted here: https://studentaffairs.duke.edu/conduct/z-policies/academic-dishonesty
